# Training Induced Changes to Skeletal Muscle Passive Properties Are Evident in Both Single Fibers and Fiber Bundles in the Rat Hindlimb

**DOI:** 10.3389/fphys.2020.00907

**Published:** 2020-08-12

**Authors:** Alex M. Noonan, Parastoo Mashouri, Jackey Chen, Geoffrey A. Power, Stephen H. M. Brown

**Affiliations:** Department of Human Health and Nutritional Sciences, University of Guelph, Guelph, ON, Canada

**Keywords:** passive mechanical properties, stiffness, passive stress, trainability, single fiber, titin, downhill running, sarcomere length

## Abstract

**Introduction:** The passive mechanical behavior of skeletal muscle represents both important and generally underappreciated biomechanical properties with little attention paid to their trainability. These experiments were designed to gain insight into the trainability of muscle passive mechanical properties in both single fibers and fiber bundles.

**Methods:** Rats were trained in two groups: 4 weeks of either uphill (UH) or downhill (DH) treadmill running; with a third group as sedentary control. After sacrifice, the soleus (SOL), extensor digitorum longus (EDL), and vastus intermedius (VI) were harvested. One hundred seventy-nine bundles and 185 fibers were tested and analyzed using a cumulative stretch-relaxation protocol to determine the passive stress and elastic modulus. Titin isoform expression was analyzed using sodium dodecyl sulfate vertical agarose gel electrophoresis (SDS-VAGE).

**Results:** Single fibers: passive modulus and stress were greater for the EDL at sarcomere lengths (SLs) ≥ 3.7 μm (modulus) and 4.0 μm (stress) with DH training compared to UH training and lesser for the SOL (SLs ≥ 3.3 μm) with DH training compared with control; there was no effect of UH training. Vastus intermedius was not affected by either training protocol. Fiber bundles: passive modulus and stress were greater for the EDL at SLs ≥ 2.5 μm (modulus) and 3.3 μm (stress) in the DH training group as compared with control, while no affects were observed in either the SOL or VI for either training group. No effects on titin isoform size were detected with training.

**Conclusion:** This study demonstrated that a trainability of passive muscle properties at both the single fiber and fiber bundle levels was not accompanied by any detectable changes to titin isoform size.

## Introduction

The ability of skeletal muscle to produce passive forces independent of activation and cross-bridge cycling is important, as passive properties affect both the amount of elastic energy stored within the muscle and the stresses that are developed at longer muscle lengths. Passive muscle force assists in resisting joint motion, the passive deformation of muscle fibers can influence cells responsible for muscle growth and repair, and excessive passive muscle loading can play a role in tendinopathies and contusion injuries (Herbert and Gandevia, 2018). The passive mechanical properties of individual fibers and bundles of fibers are known to vary between muscles (Prado et al., 2005; Ward et al., 2009; Regev et al., 2011) as well as during development (Ottenheijm et al., 2009), have been used to describe structural function (Shah et al., 2004; Prado et al., 2005; Ward et al., 2009), and have been shown to be sensitive to tissue remodeling in response to joint related injury (Safran et al., 2005; Brown et al., 2011; Sato et al., 2014; Gsell et al., 2017) and disease (Friden and Lieber, 2003; Lieber et al., 2003; Gsell et al., 2017), yet these passive muscle properties are generally underappreciated.

At the whole muscle level, it is generally recognized that the extracellular matrix has a large role in dictating muscle passive properties (Prado et al., 2005; Gillies and Lieber, 2011; Meyer and Lieber, 2018; Ward et al., 2020); however others have argued that the intracellular protein titin is predominantly responsible for whole muscle passive stiffness at short (2.4–2.7 μm) sarcomere lengths (SLs; Brynnel et al., 2019). At the single fiber level, it is generally thought that titin is primarily responsible for passive stiffness and tension (Wang et al., 1979; Horowits et al., 1986; Trombitás et al., 1995; Granzier et al., 1996, 2003; Linke et al., 1996; Kontrogianni-Konstantopoulos et al., 2009); however, the contribution of other intra/extra cellular proteins should not be excluded (Ramsey and Street, 1940; Rapoport, 1973; Tidball, 1986; Shah et al., 2004; Noonan et al., 2020a). While these mechanistic studies have aimed to elucidate the relative roles of the intracellular and extracellular matrix proteins in dictating passive muscle properties, little attention has been paid to the trainability of skeletal muscle passive properties at both the single fiber and fiber bundle levels.

Several studies have examined the effect of both eccentric (ECC) (Nelson and Bandy, 2004; Kay et al., 2016) and isometric (ISO; Klinge et al., 1997) muscle training on *in vivo* joint stiffness and range of motion in humans. While several ECC-based training protocols report an increased joint range of motion and stretch tolerance (Nelson and Bandy, 2004; Kay et al., 2016), others have found a decrease in joint compliance (i.e., an increase in passive joint stiffness Klinge et al., 1997) with ISO-based training. Though these studies provided insight into training effects on joint range of motion, they were limited in their ability to isolate possible mechanisms at the level of individual muscles.

In animals, the effects of treadmill-specific training on whole muscle passive stiffness are unclear. While Kovanen et al. (1984) found that the passive stiffness of the predominantly slow twitch soleus (SOL) increased significantly with level-ground treadmill training, the predominantly fast twitch rectus femoris demonstrated no change in stiffness. In contrast, Reich et al. (2000) demonstrated an increase in the passive lengthening force of the long head of the triceps brachii muscle (predominately fast twitch) with ECC-focused treadmill training; clearly, more work is needed to clarify possible muscle adaptations with these types of training.

Therefore, the purpose of these experiments was to gain additional insight into the trainability of muscle passive mechanical properties at both the single muscle fiber and fiber bundle levels. To do this we employed a model of uphill (UH) and downhill (DH) treadmill running in rats to investigate changes in passive muscle properties in single fibers and fiber bundles across a wide range of SLs (1.7–4.0 μm).

## Materials and Methods

### Animals

Thirty male CD® Sprague Dawley IGS rats (sacrificial age: 18.8 ± 0.28 weeks and mass: 523.6 ± 11.0 g) were obtained (Charles River Laboratories, Senneville, QC, Canada). The study was performed with approval from the Animal Care Committee of the University of Guelph. Rats were housed in groups of three and free fed a Teklad global 18% protein rodent diet (Envigo, Huntingdon, Cambridgeshire, UK) and water. Each rat was acclimatized for 1 week to the new housing conditions, then familiarized with treadmill running, and assigned to one of the three experimental groups: UH running, DH running, and sedentary control (i.e., no running intervention). Following 20 days of exercise, rats recovered for 72 h before sacrifice *via* CO_2_ asphyxiation followed by cervical dislocation for experimental testing.

### Training Protocol

One week prior to training, rats were familiarized with treadmill running (on a 0° grade). Rats in the exercise intervention groups (i.e., UH or DH running) were run 5 days/week on an EXER 3/6 animal treadmill (Columbus Instruments, Columbus, OH, USA) set to a 15° incline or decline for 20 training days over a 4 week period. Training sessions lasted 15 min on day 1 and the daily duration was increased by 5 min/day to the target 35 min (by day 5) for the remainder of the training period. At the start of each training session, rats started at a walking speed of 10 m/min, which was gradually increased to 16 m/min, at a rate of 1 m/min. Each training session was delivered in a series of 5 min bouts (i.e., 3 on day 1 and 7 on days 5 to the end of the 4 week period) with 2 min or rest between each bout (Chen et al., 2020).

### Experimental Procedure

After sacrifice, extensor digitorum longus (EDL), Soleus (SOL), and vastus intermedius (VI) muscles were dissected. Part of the muscle was immediately placed into physiological storage solution (Eastwood et al., 1979) and stored at 4°C for 24 h and subsequently placed into fresh storage solution and stored at −20°C for at least 1 week (maximum 1 month) prior to mechanical testing. This was done to permeabilize the sarcolemma as well as to prevent breakdown of the sample (Shah and Lieber, 2003). Another part of the muscle was frozen at −80 for titin protein analysis.

**Figure 1:**
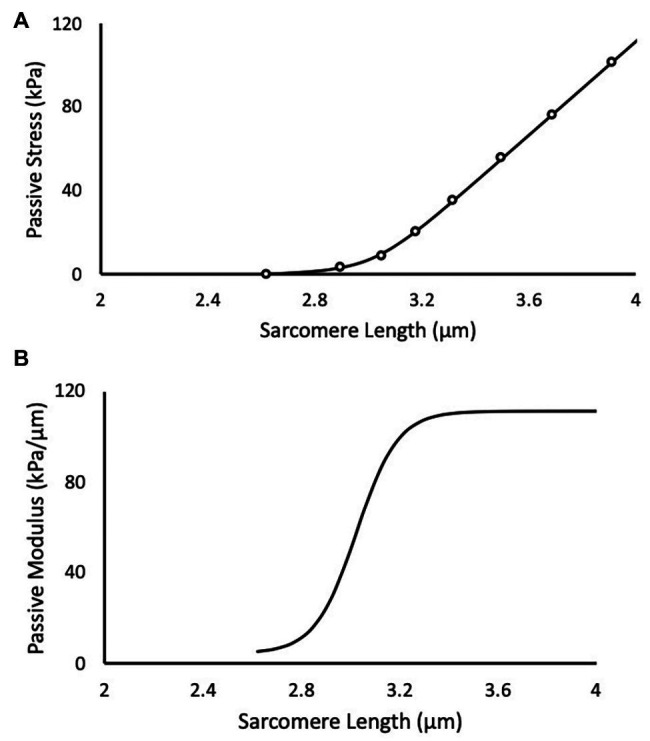
A representative experimental test of a muscle fiber bundle is depicted in black. **(A)** Scatter points are experimental data and solid line is the predicted fit of the logistic integral. **(B)** The corresponding passive elastic modulus-sarcomere length (SL) relationship for the test.

Mechanical testing was performed in a relaxing solution consisting of (in mM) 59.4 imidazole, 86 KMSA, 0.13 Ca (MSA)2, 10.8 Mg (MSA)2, 5.5 K3 EGTA, 1 KH_2_PO_4_, 0.05 leupeptin, and 5.1 Na_2_ATP (Shah and Lieber, 2003). Typically, two single muscle fibers and two bundles of fibers (8–20 fibers ensheathed in their extracellular matrix) were dissected and tested from each muscle. Dissection was performed in a relaxing solution, where fiber bundles were first separated and either tested or further dissected into single fibers. The single fiber or fiber bundle were then tied at either end to two separate pins: one attached to a micro-level force transducer (Model-405A, Aurora Scientific Inc., Aurora, Ontario, Canada) and the other to a high-speed motor (Model-322C, Aurora Scientific Inc., Aurora, Ontario, Canada). Samples were set to their slack length (length at which passive resistance to stretch was first detected), and measurements of the diameter were taken at three locations along the fiber or bundle length using a digital micromanipulator (precision 1 μm). The fiber or bundle was trans-illuminated by a 5-mW diode laser (Coherent, Wilsonview, Oregon, USA) and the resultant diffraction pattern was used to calculate SL (Lieber et al., 1984, p. 146). Force and SL were recorded as fibers and bundles were rapidly stretched in progressive increments of ~0.25 μm/sarcomere and allowed to relax for 2 min. The force at the end of each 2-min period was normalized to the cross-sectional area calculated from the average of the diameter measures (assuming a cylindrical shape) to give a value of stress (Ward et al., 2009). For each test, a minimum of five sequential stretches was required for the test to be considered successful. Here, the passive elastic modulus (*M* = derivative of the stress-sarcomere length relationship) was assumed to follow a logistic function (Zwambag et al., 2019) of the form

(1) $$M\left(l\right)=\left(\frac{\Delta M}{1+e^{-k\left(l-t\right)}}+M_{S}\right)$$

for all SLs (l) greater than slack length (Figure 1). This conceptual model assumes that the initial modulus at slack length (*M_S_*) increases by ΔM. The coefficients *k* and *t* are rate and length constants, respectively, and they determine the smooth transition between initial and final moduli. The mechanical characteristics (*M_S_*,ΔM, *k*, and *t*) of each single muscle fiber were determined by fitting the integral of equation (1)

(2) $$\sigma \left(l\right)=\frac{\Delta M}{k}ln\left(1+e^{k\left(l-t\right)}\right)+M_{S}l+C$$

to the stress-SL data using a non-linear least-squares optimizer (MATLAB 2014b) and a Trust-Region-Reflective algorithm. The coefficient *C* is an arbitrary integration constant. Equations 1 and 2 were evaluated in 0.1 μm SL increments between 1.7 and 4.0 μm for each muscle fiber or bundle to determine an effect of training and length on passive elastic modulus and passive stress.

### Titin Analysis

Titin protein isoforms were analyzed in a subset of animals (*n* = 4 per group) by SDS-VAGE (Warren et al., 2003). Frozen samples (~30 mg) from the EDL and SOL were homogenized in solubilization buffer [61 mM Tris, 2.78% SDS, 5% 2-B mercaptoethanol, 11% glycerol, 0.02% (w/v), bromophenol blue, and 4 μg/ml leupeptin (pH 6.8)] at a ratio of approximately 0.03 μg muscle mass/μl buffer. Titin standards from rat cardiac (~3,000 kDa) and rat gastrocnemius (~3,600 kDa; Li et al., 2012) were also homogenized and mixed to create a standard cocktail. Samples were incubated for 5 min on ice and then heated for 10 min at 60°C. Protein bands were visualized with a Coomassie G-250 stain. Gels were then digitized and analyzed (FluoraChem HD2 chemiluminescent, Alpha Inotech, Santa Clara, US) for their molecular mass, which was based on the mobility of the experimental bands relative to the rat cardiac and gastrocnemius titin standards. Molecular mass was compared among training groups from the same muscle.

### Statistical Analysis

The mechanical testing dependent variables (passive elastic modulus and passive stress at SLs between 1.7 and 4.0 μm in increments of 0.1 μm) were compared using mixed model ANOVAs (SAS Institute, Cary NC, USA). The fixed effect was training group (Control vs. UH vs. DH) with a random effect of animal to account for repeated sampling. This type of analysis has been shown to best represent the biomechanical variability in a hierarchal dataset (Tirrell et al., 2018). Titin molecular mass from each muscle homogenate was compared between groups from within the same muscle using a one-way ANOVA. All data are reported as means ± standard errors (SEM).

## Results

A total of 364 (179 bundles and 185 fibers) tests were performed and analyzed.

### Single Muscle Fiber Passive Modulus

**Figure 2:**
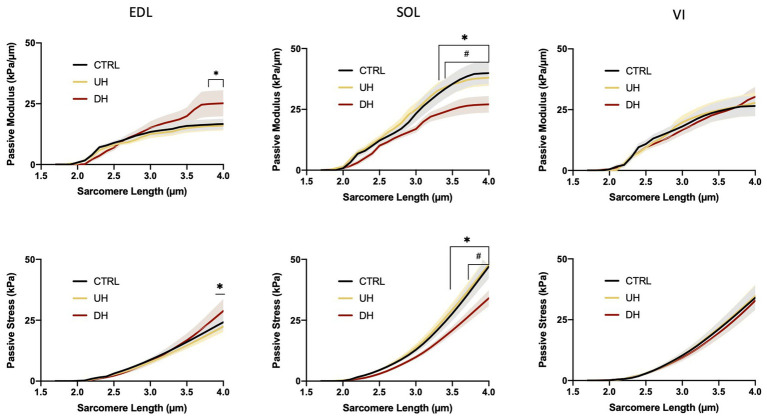
Mean (solid lines) and SEM (shaded) single muscle fiber passive elastic modulus (**top panel**) and stress (**bottom panel**) for each muscle [extensor digitorum longus (EDL), soleus (SOL), and vastus intermedius (VI)] across the range of SLs. ^*^indicates statistical difference between DH and UH groups. ^#^indicates statistical difference between control (CTRL) and downhill (DH) groups. (*n* = 185; *p* < 0.05).

There was an effect of training group on the EDL and SOL but not the VI (Figure 2). Specifically, for the EDL, there was a significantly higher passive elastic modulus in the DH group compared to the UH group at SLs between 3.7 and 4.0 μm (value of *p* range across these SLs: *p* = 0.0169–0.0192; Figure 2) but no differences were found between the DH and control group. For the SOL, the passive elastic modulus was significantly lower in the DH compared to the UH group at SLs between 3.3 and 4.0 μm (*p* = 0.0172–0.0347), and in the DH compared to the control group at SLs between 3.4 and 4.0 μm (*p* = 0.0043–0.0351; Figure 2).

### Single Muscle Fiber Passive Stress

There was an effect of training group on the EDL and SOL but not the VI (Figure 2). Specifically, for the EDL, the passive stress was higher in the DH compared to the UH group at the longest SL tested (4.0 μm; *p* = 0.0251; Figure 2), but no differences were found between the DH and control group. For the SOL, passive stress was lower in the DH group compared to the UH group at SLs between 3.4 and 4.0 μm (*p* < 0.0001–0.0348), and in the DH group compared to the control group at SLs between 3.7 and 4.0 μm (*p* = 0.0002–0.0487; Figure 2).

### Muscle Bundle Passive Modulus

**Figure 3:**
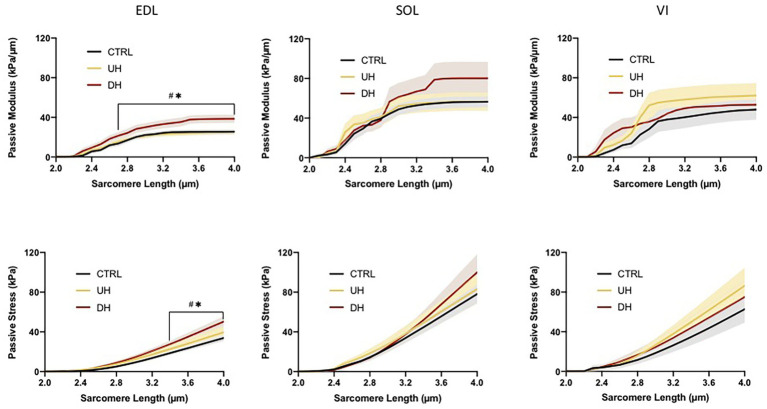
Mean (solid lines) and SEM (shaded) muscle fiber bundle passive elastic modulus (**top panel**) and stress (**bottom panel**) for each muscle (EDL, SOL, and VI) across the range of SLs. ^*^indicates statistical difference between DH and uphill (UH) groups. ^#^indicates statistical difference between CTRL and DH groups. (*n* = 179; *p* < 0.05).

There was an effect of training group for the EDL muscle; there were no group differences for either the SOL or the VI (Figure 3). For the EDL, the passive elastic modulus was higher in the DH group compared to both the UH group and control group between SLs of 2.5 and 4.0 μm [*p* = 0.0040–0.0155 (DH vs. control (CTRL)) and *p* = 0.0032–0.0486 (DH vs. UH); Figure 3].

### Muscle Bundle Passive Stress

There was an effect of training group for the EDL but not for the SOL or VI (Figure 3). Specifically, the EDL had significantly higher passive stresses in the DH group compared to the UH and control groups at SLs between 3.3 and 4.0 μm [*p* < 0.0001–0.0296 (DH vs. CTRL) and *p* < 0.0001–0.0385 (DH vs. UH); Figure 3].

### Titin Isoform Size

**Figure 4:**
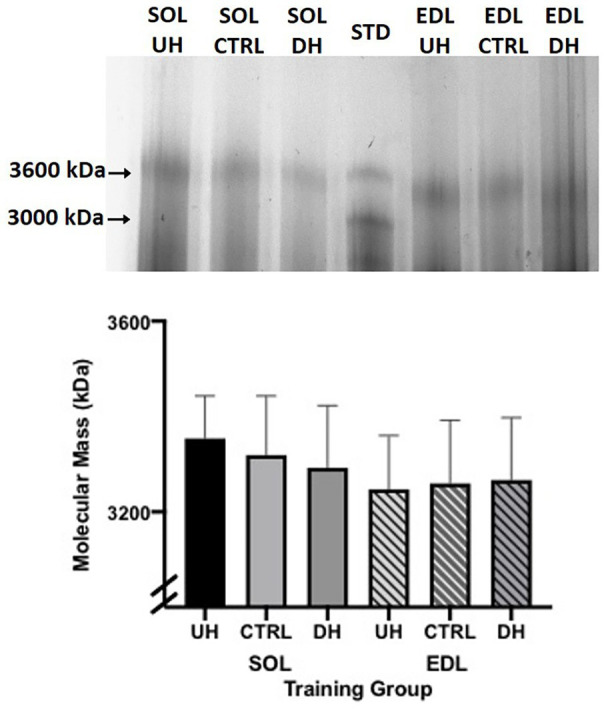
**Top panel**: example sodium dodecyl sulfate vertical agarose gel electrophoresis (SDS-VAGE) gel showing titin bands for SOL and EDL UH, CTRL and DH groups, and standard (STD) lane containing rat cardiac and gastrocnemius. **Bottom panel**: mean (+SEM) titin molecular mass (kDa) following 4 weeks of treadmill training and sedentary control. Solid bars (SOL) and striped lines [EDL; *n* = 4; *p* = 0.31 (SOL) and *p* = 0.53 (EDL)].

No measurable difference was observed in the titin isoform size between any of the groups (DH, UH, and sedentary control) for either the SOL (*p* = 0.3063) or EDL (*p* = 0.5330; Figure 4).

## Discussion

This study was designed to provide insight into the trainability of the passive mechanical properties of muscle. A model of UH and DH treadmill running was employed in rats to investigate changes in muscle passive mechanical properties at both the single fiber and fiber bundle levels across a wide range of SLs (1.7–4.0 μm). The novel findings from this study are that (1) training *via* DH running caused differential changes (increased and decreased stiffness in EDL and SOL, respectively) in single fibers from predominantly slow (SOL) and fast (EDL) muscle, (2) training *via* DH running increased the stiffness of fiber bundles in the EDL, and (3) changes at both the single fiber and fiber bundle level were length dependent; specifically, all statistically significant changes occurred at SLs equal to or greater than 2.5 μm and most occurred at longer (≥3.3 μm) SLs. Finally, no detectable differences in the titin isoform size between training groups (DH, UH, and control) were found.

To provide context to these results, some knowledge of the mechanical action of the muscles studied during treadmill running is helpful. It is generally accepted that the anti-gravity plantarflexors (including SOL) and knee extensors (including VI) are loaded primarily eccentrically during DH running and concentrically during UH running (Margaria, 1968; Armstrong et al., 1983; Butterfield et al., 2005; Maeo et al., 2017). Separate work on the animals tested in the current study (Chen et al., 2020) demonstrated that the EDL had a greater number of sarcomeres in series in both DH and UH training groups compared to the sedentary control group, which may suggest that the EDL performed meaningful eccentric contractions during both DH and UH running.

### Single Muscle Fiber Passive Elastic Modulus and Stress

To the authors’ knowledge, the current study is the first to demonstrate training induced changes to the passive mechanical properties of skeletal muscle at the single fiber level. Specifically, DH training induced a length dependent increase in the passive modulus and stress of the fast type EDL when compared to UH running, a length dependent decrease in the passive modulus and stress in the slow type SOL when compared to both UH and CTRL groups, and no change with either DH or UH training in the mixed type VI muscle; all differences were observed at SLs ≥3.3 μm. Previous studies have shown that the passive mechanical properties of single muscle fibers are susceptible to change with age (Lim et al., 2019; Noonan et al., 2020b), in response to joint related injury (Safran et al., 2005; Brown et al., 2011; Sato et al., 2014; Gsell et al., 2017), and disease (Friden and Lieber, 2003; Lieber et al., 2003; Gsell et al., 2017), so it is not surprising that these properties can also be altered in response to treadmill training.

Titin is generally considered to be the primary determinant of single fiber passive mechanical properties (Magid and Law, 1985; Bartoo et al., 1997; Prado et al., 2005; Brynnel et al., 2019). However, in the current study, no measurable difference in titin isoform size between training groups was detected. While the number of animals used to measure titin size was limited (*n* = 4 per group), any trends in size differences were actually opposite to what would be expected to explain the passive mechanical property results. For example, in the DH group compared to control the passive elastic modulus and stress were greater and lesser in the EDL and SOL, respectively. For titin to explain these results, we would expect smaller (stiffer) and larger (less stiff) titin isoforms in the EDL and SOL, respectively; however, any non-statistically significant trends in the titin data demonstrate the opposite. Therefore, we believe that differences in titin isoform size do not explain the observed training effects on passive mechanical properties. Previous studies that have examined titin isoform size after exercise have shown mixed results. In humans, no change in the relative titin isoform content and/or isoform expression (size) in human lower limb muscle has been reported following eccentric exercise training (Ochi et al., 2006), jump squat training (McGuigan et al., 2003), or plyometric training (Pellegrino et al., 2016). In partial contrast to these findings, Trappe et al. (2002) found titin to be significantly degraded 24 h after a single bout of high intensity eccentric training; however, it is unknown if titin isoform size or content returned to normal following a recovery period or if it was permanently changed. In rodents, Hidalgo et al. (2014) demonstrated increases in skeletal muscle (diaphragm) titin size (with no concurrent functional measurements) with free wheel running (Hidalgo et al., 2014), while Muller et al. (2014) reported increased cardiomyocyte passive stiffness that was associated with varied parts of the titin protein undergoing phosphorylation in response to acute training on a wheel.

Thus, it seems that the response of titin to exercise training varies – possibly dependent on the duration of exercise, duration of training program, exercise type, and muscle types analyzed. The current study demonstrated no alterations to titin isoform size in response to chronic treadmill training despite changes in single fiber passive stress and stiffness at the cellular level. Most studies concerning titin’s role on passive muscle properties are performed at SLs on the ascending and plateau region of the active force-length curve (Muller et al., 2014; Brynnel et al., 2019). However, the changes in the passive elastic modulus and stress observed in the current work occurred at SLs ≥ 3.3 μm (SOL) and 3.7 μm (EDL). At these lengths, the collagen-based extracellular matrix (ECM) components surrounding the cell have also been implicated in determining single fiber passive mechanical properties (Ramsey and Street, 1940; Rapoport, 1973; Tidball, 1986; Noonan et al., 2020a); therefore, it is possible that the changes observed with training in the current study are owed to remodeling of the ECM network surrounding the muscle fiber. However, titin modifications are a complex biological process (Ottenheijm et al., 2009) and as such further work is needed to define both its role and the ECM’s role in the functional adaptations to exercise.

### Muscle Bundle Passive Modulus and Stress

Only limited studies have explored differences in passive mechanical properties with training at the whole muscle level (Kovanen et al., 1984; Reich et al., 2000). The current study revealed that differences in muscle fiber bundle passive properties were observed with DH training but not UH training compared to control and that these differences were limited to the fast type EDL muscle (no statistically significant changes were observed in the slow type SOL muscle or the mixed type VI muscle). The novelty of these findings demonstrate that training-induced differences in muscle fiber bundle passive properties are most apparent in DH training, fast type (EDL) muscles, and are only apparent at SLs at or above 2.5 μm (approximate active optimal length for rat muscle), when moduli and stresses become more substantial.

The current findings align with those of Reich et al. (2000) who demonstrated an increase in the passive lengthening force of the long head of the triceps brachii muscle (a predominately fast type muscle Delp and Duan, 1996) with ECC training in rats. In contrast to our findings, Kovanen et al. (1984) found that the passive stiffness of the slow muscle (SOL) increased significantly with level-ground treadmill training but found no changes in the predominantly fast rectus femoris. It should be noted that Kovanen et al. (1984) employed level-ground treadmill training (no incline or decline) and only measured stiffness in a small linear region of the force-deformation curve and were therefore unable to fully capture the entire passive stiffness/stress length curve. Further, the fast rectus femoris has a different functional role than the fast EDL; rectus femoris is part of the quadriceps group, and the quadriceps muscle examined in the current study also showed no affect with training. Therefore, it is quite possible that the functional role/action of the muscle group is a more important factor in determining its adaptation to training than its fiber type distribution.

In addition, similar findings to ours in rabbits (Ducomps et al., 2003) and rats (Pousson et al., 1991) suggest that jump training can increase muscle stiffness in fast type and mixed muscles (EDL and rectus femoris), whereas no change (Ducomps et al., 2003) or a decrease (Pousson et al., 1991) was found in slow type (SOL) muscles; these stiffness changes were correlated with increases and decreases, respectively, in collagen concentration with exercise training. At the whole muscle and muscle bundle level, the muscle is held together by the collagen-based ECM, which is generally considered to be the main determinant of passive mechanical properties at these levels (Prado et al., 2005; Gillies and Lieber, 2011; Meyer and Lieber, 2018; Zwambag et al., 2019). Therefore, the statistically significant changes in the passive elastic modulus, which were apparent at shorter lengths in the bundles but not the fibers (as bundles contain relatively more extracellular matrix than single fibers; Ward et al., 2020), suggest that collagen remodeling may be one source of the training-induced changes observed in the current study. Future work will need to explore this further.

It is important to note that in the muscle fiber bundles, both EDL and soleus demonstrated similar magnitude mean increases in passive elastic modulus and stress in DH compared to UH and control; however, the variability was much larger in the SOL, and therefore, these increases were not statistically significant (values across the range of SLs of the modulus of *p* = 0.1282–0.9020; stress values of *p* = 0.1582–0.9558). It has been found that slow-twitch muscles of rats contain more collagen in the ECM than fast-twitch muscles (Zimmerman et al., 1993; Ahtikoski et al., 2003), which may be related to the more variable response in the soleus fiber bundles observed here. It is not entirely clear why there were no effects of training observed in the mixed type VI muscle, although evidence suggest that not all regions of the VI are loaded equally during DH running (Maeo et al., 2017). As such, it is possible that in the current study, the specific muscle fibers/bundles tested were from regions not particularly affected by DH or UH training.

Taken together, the results of both the muscle fiber bundle and single fiber analyses indicate that functional changes in the passive modulus and stress occur in a length dependent manner, which is most prominent at longer SLs (≥2.5 μm in fiber bundles and ≥3.3 μm in single fibers). Although the mechanisms behind these functional changes are not completely known, it is possible that changes at the bundle and single fiber level are both a physiological adaptation as well as a possible protective mechanism. While the exact interplay between different muscles and bundles and fibers is not fully understood, it is likely that the adaptation is based on a combination of factors, including muscle action, usage, and composition (fiber type distribution, collagen content, and baseline mechanical properties). Functionally, the increase (EDL) and decrease (SOL) in the passive modulus and stress in single fibers, and the increase (EDL) in passive modulus and stress at the bundle level would lead to altered loading and energy storage capabilities within the muscles. Whether or not these altered properties are beneficial or detrimental is currently not known. However, this study provides evidence that exercise training on a treadmill can alter muscle passive mechanical properties at both the fiber bundle and single fiber levels. This could have implications for treating detrimental changes to muscle properties that occur with aging (Lim et al., 2019) and disease (Friden and Lieber, 2003; Lieber et al., 2003; Gsell et al., 2017).

## Data Availability Statement

The datasets generated for this study are available on request to the corresponding author.

## Ethics Statement

The animal study was reviewed and approved by University of Guelph Animal Care Committee.

## Author Contributions

All authors designed the study. AN, JC, and PM performed training of the animals. AN and PM collected muscles. AN conducted the experiments. AN and SB performed the data analysis. All authors contributed to the article and approved the submitted version.

### Conflict of Interest

The authors declare that the research was conducted in the absence of any commercial or financial relationships that could be construed as a potential conflict of interest.
